# Predisposition to Lung Adenocarcinoma in a Family Harboring the Germline *EGFR* V843I Mutation

**DOI:** 10.1200/PO.19.00104

**Published:** 2019-07-26

**Authors:** Kouki Ohtsuka, Hiroaki Ohnishi, Masachika Fujiwara, Takeshi Morii, Satsuki Matsushima, Wataru Ogura, Satoko Yamasaki, Tomonori Kishino, Ryota Tanaka, Takashi Watanabe

**Affiliations:** ^1^ Kyorin University School of Medicine, Tokyo, Japan

## INTRODUCTION

Mutations in the tyrosine kinase domain of epidermal growth factor receptor (*EGFR*), most commonly a deletion in exon 19 or an L858R substitution in exon 21, are frequent in patients with non–small-cell lung cancer. These *EGFR* mutations are speculated to constitutively activate EGFR through phosphorylation and impart tumorigenic properties.^1^ Most *EGFR* mutations occur in somatic tumor tissue, with germline *EGFR* mutations being extremely rare.^2,3^ As a scarce example, the germline *EGFR* T790M or germline V843I mutation has been identified in several families susceptible to lung cancer.^2-6^

We previously reported multiple cases of lung adenocarcinoma in a family with the germline *EGFR* V843I mutation.^5^ The proband had advanced-stage cancer resistant to several treatments, including EGFR–tyrosine kinase inhibitors, resulting in poor therapeutic outcomes. In contrast, two other family members diagnosed with early-stage lung adenocarcinoma achieved long-term relapse-free survival after surgery without additional treatment. However, tumors of the proband and other family members both harbored the same somatic *EGFR* L858R mutation in addition to the germline V843I mutation, and it is unclear how these two genetic mutations affected the prognosis of lung cancer in those patients. Furthermore, germline mutations causing hereditary cancers other than the *EGFR* V843I mutation may prevail in this family, because only *EGFR* mutations were previously assessed in this family. Therefore, we performed whole-genome sequencing (WGS) and target sequencing (TS) of oncogenes in cancerous and normal tissues of family members to determine whether they harbored germline mutations and/or somatic oncogenic mutations, other than the *EGFR* mutation, associated with cancer pathogenesis and prognosis.

## CASE REPORT

The proband was a 48-year-old Japanese woman with stage IV lung papillary adenocarcinoma, T4N2M1 with pleural dissemination. Despite various treatments including EGFR–tyrosine kinase inhibitors, she died as a result of progressive lung cancer within 1 year and 6 months of treatment initiation. The proband’s mother underwent lobectomy at 61 years of age for stage IA lung papillary adenocarcinoma. She is alive and disease free 22 years and 4 months after surgery. The proband’s younger brother was diagnosed with stage IA lung papillary adenocarcinoma at 41 years of age and underwent lobectomy. He is alive and disease free 9 years and 3 months after surgery. The proband’s aunt had lung cancer at 70 years of age; however, no precise data are available regarding her disease course and prognosis. The proband’s nephew had cecal non-Hodgkin lymphoma at 12 years of age and is alive and disease free after surgery and intensive chemotherapy. No other family history of malignant disease was revealed on a detailed interview (Table 1).

**TABLE 1.**
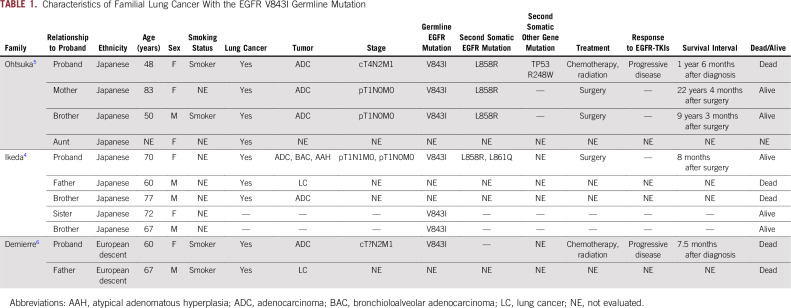
Characteristics of Familial Lung Cancer With the EGFR V843I Germline Mutation

### Sample Preparation

The study was approved by the ethics committee of the participating institutes, and written informed consent was obtained from the proband and two family members with lung cancer. Peripheral blood mononuclear cells of the proband, cancerous pleural effusion from the proband, and formalin-fixed paraffin-embedded tumor samples from her mother and younger brother were subjected to genetic analysis. Genomic DNA was extracted from these samples for next-generation sequencing (NGS) analysis using the DNeasy Blood & Tissue kit (Qiagen, Hilden, Germany).

### WGS Analysis

We performed WGS using genomic DNA extracted from cancer cells harvested from the cancerous pleural effusion and compared it to that of whole blood. Paired-end sequencing was performed using Illumina’s NGS platforms HiSeq X Ten system (Illumina, San Diego, CA). Sequence reads were aligned against the reference human genome (hg19) with Burrows-Wheeler Aligner. Single-nucleotide variants and insertions/deletions in cancer tissue and normal blood genomes were identified using GATK and SomaticSniper. Copy number variations were analyzed using Control-FREEC. Structural variations identified using both BreakDancer and Pindel were further analyzed. We adopted driver gene mutations registered in the Catalogue of Somatic Mutations in Cancer (COSMIC) database, predicted functional consequences using SIFT and PolyPhen-2 software, or categorized them as pathogenic or likely pathogenic per the ClinVar database. Furthermore, we examined known causative germline mutations of hereditary cancers and somatic mutations of major cancer-related genes among the gene alterations in the proband.

### TS Analysis

For TS analysis, we used the Ion AmpliSeq Custom Panel and Ion Torrent PGM deep sequencing Ion AmpliSeq Cancer Hotspot Panel v2 (Thermo Fisher Scientific, Waltham, MA). Barcoded libraries were pooled and sequenced on Ion 318 Chip using Ion Torrent PGM in accordance with the manufacturer’s instructions. We examined mutations in 50 cancer-related genes in tumor DNA derived from the proband and two of her family members.

### Genomic Study

WGS revealed the previously reported germline *EGFR* V843I mutation; however, no other germline oncogenic mutations were observed in the peripheral blood and cancerous pleural effusion of the proband. Furthermore, WGS revealed a de novo *TP53* R248W somatic mutation in addition to a second *EGFR* mutation, L858R, in the cancer cells of the proband. The *EGFR* V843I and L858R mutations and the *TP53* R248W mutation have been registered in the COSMIC database as functional mutations per the SIFT and PolyPhen-2 software. In the ClinVar database, the *EGFR* V843I mutation has been registered as likely pathogenic, and the *TP53* R248W mutation is pathogenic. Other genetic abnormalities, including insertions/deletions, copy-number variations, and structural variations of cancer-related genes, were not detected in blood or cancer cells of the proband (Tables 1 and 2). TS analysis of mutations in 50 cancer-related genes revealed the same mutations as those confirmed via WGS analysis of cancer cells of the proband. In contrast, only the *EGFR* V843I and L858R mutations were identified in tumors in the mother and brother of the proband. *TP53* mutations including R248W identified in cancer tissue of the proband were not detected in that of her mother and brother (Table 3).

**TABLE 2.**
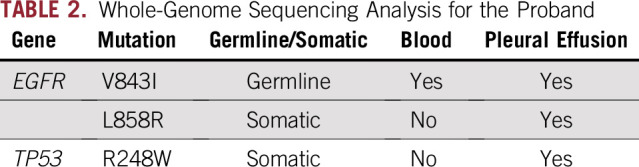
Whole-Genome Sequencing Analysis for the Proband

**TABLE 3.**
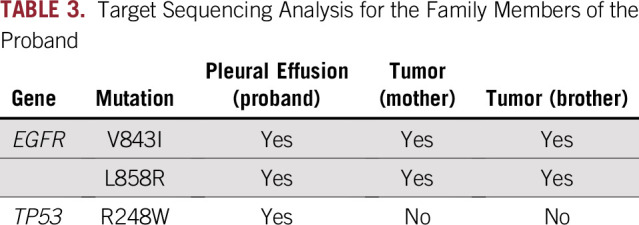
Target Sequencing Analysis for the Family Members of the Proband

## DISCUSSION

We previously hypothesized that multiple occurrences of lung adenocarcinoma within the family are associated with the inherited germline *EGFR* V843I mutation and acquired somatic L858R mutation.^5^ Furthermore, functional analysis of the *EGFR* V843I mutation revealed that this mutation has transforming and proliferative ability.^7^ However, unidentified germline mutations causing hereditary cancers other than the *EGFR* V843I mutation are possibly associated with cancer predisposition in this family, because only *EGFR* mutations were analyzed previously.^5^ To our knowledge, no study has investigated risk-associated germline mutations for hereditary cancers other than *EGFR* via WGS analysis in families harboring the germline *EGFR* mutation (Table 1).^4-6^ WGS analysis of peripheral blood samples of the proband revealed no known genetic abnormalities for hereditary cancers other than the germline *EGFR* V843I mutation, further supporting the possibility that this mutation causes familial lung adenocarcinoma (Tables 1 and 2). According to the Tohoku University Tohoku Medical Megabank Organization database, the frequency of the *EGFR* V843I germline mutation is low, amounting to one in 3,509 healthy Japanese individuals; however, it is considered an important germline mutation associated with the risk of lung carcinogenesis.^8,9^

Furthermore, the somatic *TP53* R248W mutation was detected only in cancer cells of the proband but not the other two family members (Tables 2 and 3). TP53 promotes tumorigenesis in various cancers, including lung cancer. *TP53* mutations have been frequently reported in lung adenocarcinomas, with a prevalence of 39% and 52% per the COSMIC and The Cancer Genome Atlas databases, respectively. In this study, a *TP53* mutation was detected only in the proband presenting with a poor prognosis and minimal effects of anticancer therapy. In contrast, the proband’s mother and brother, both lacking this mutation, achieved long-term relapse-free survival after surgery (Table 1). The other 48 cancer-related oncogenes assessed via TS analysis were not mutated in tumors of any of the family members. Although this is an anecdotal case, the present results suggest that the *TP53* mutation may serve as a prognostic factor predicting worse drug sensitivity and poor therapeutic outcomes in lung cancer harboring a germline *EGFR* mutation. Recent studies have reported that lung cancers with both *EGFR* and *TP53* mutations are associated with a poor prognosis.^10-12^ The present findings support our hypothesis that lung cancers harboring *EGFR* and *TP53* mutations are refractory and have a poor prognosis, suggesting that analysis of tumor-related oncogenes via WGS or TS may help predict the clinical course of familial lung cancer cases.

Furthermore, mutations in genes other than *EGFR* are reportedly associated with familial accumulation of lung cancer, including germline *RB1*, *HER2*, or *TP53* mutations.^3,13^ Among these, Li-Fraumeni syndrome, characterized by germline *TP53* mutations, is potentially the most frequent multiple cancer syndrome associated with an increased risk of lung cancer.^3^ High-throughput analysis of genes associated with multiple cancers, particularly *TP53*, is therefore crucial to elucidate the genetic background of patients with familial lung cancer. Considering the high prevalence of *TP53* mutations, somatic or germline, in cases of solitary or hereditary lung cancer, *TP53* mutations are apparently an equally prominent cause of lung cancer as *EGFR* mutations.

In conclusion, NGS analysis of the genome of family members with the germline *EGFR* V843I mutation reinforced the hypothesis that this mutation predisposes individuals to familial lung adenocarcinoma. The acquired *TP53* R248W mutation is potentially associated with a poor prognosis in the proband in the lung cancer–predisposed family.
